# Propolis anti-inflammatory effects on MAGE-1 and retinoic acid-treated dendritic cells and on Th1 and T regulatory cells

**DOI:** 10.1590/1678-9199-JVATITD-2022-0044

**Published:** 2023-01-13

**Authors:** Karina Basso Santiago, Bruno José Conti, Eliza de Oliveira Cardoso, Fernanda Lopes Conte, Karen Ingrid Tasca, Graziela Gorete Romagnoli, Marjorie de Assis Golim, Maria Tereza Cruz, José Maurício Sforcin

**Affiliations:** 1 Institute of Biosciences, São Paulo State University (UNESP), Botucatu, SP, Brazil.; 2 School of Medicine (FMB), São Paulo State University (UNESP), Botucatu, SP, Brazil.; 3 Faculty of Pharmacy, Center for Neurosciences and Cellular Biology, University of Coimbra, Coimbra, Portugal.

**Keywords:** Dendritic cells, T CD4^+^ cells, MAGE-1, Retinoic acid, Propolis, Immunomodulation

## Abstract

**Background:**

Propolis exhibits huge potential in the pharmaceutical industry. In the present study, its effects were investigated on dendritic cells (DCs) stimulated with a tumor antigen (MAGE-1) and retinoic acid (RA) and on T lymphocytes to observe a possible differential activation of T lymphocytes, driving preferentially to Th1 or Treg cells.

**Methods:**

Cell viability, lymphocyte proliferation, gene expression (T-bet and FoxP3), and cytokine production by DCs (TNF-α, IL-10, IL-6 and IL-1β) and lymphocytes (IFN-γ and TGF-β) were analyzed.

**Results:**

MAGE-1 and RA alone or in combination with propolis inhibited TNF-α production and induced a higher lymphoproliferation compared to control, while MAGE-1 + propolis induced IL-6 production. Propolis in combination with RA induced FoxP3 expression. MAGE-1 induced IFN-γ production while propolis inhibited it, returning to basal levels. RA inhibited TGF-β production, what was counteracted by propolis.

**Conclusion:**

Propolis affected immunological parameters inhibiting pro-inflammatory cytokines and favoring the regulatory profile, opening perspectives for the control of inflammatory conditions.

## Background

Propolis is produced by honeybees from different parts of plants and presents possible applications in the pharmaceutical and food industry [1-5]. It has been used in folk medicine for centuries due to its medicinal properties. Incas used propolis as an antipyretic agent; Romans and Greeks used it for treating wounds [6]. There are different types of propolis in Brazil such as green, red and brown and their pharmacological properties may vary according to their chemical composition, which is complex and depends on the botanical source and geographical location where they were produced. Propolis composition may include aromatic aldehydes, amino acids, fatty acids, diterpenes, sesquiterpenes, esters, lignans, alcohols, vitamins, and minerals [2, 6]. 

Propolis anti-inflammatory action has been investigated both *in vitro* and *in vivo* [7, 8]. Propolis may exert pro- or anti-inflammatory activity depending on concentration, intake period and experimental conditions, affecting mechanisms involved in the inflammatory/immune response such as neutrophil adhesion and transmigration, cytokines, chemokines, prostaglandin E2, C-reactive protein, and signaling pathways [9-12]. 

Innate immunity is involved in the recognition of pathogens, leading to inflammatory responses. The sensing of microbes by receptors expressed in antigen presenting cells (APCs) such as dendritic cells (DCs) induces the activation of adaptive immunity [13].

The acquired immune response is regulated by cytokines that determine the lymphocyte profile generated after T cell activation and differentiation: Th1 cells are characterized by differentiation of T *naïve* cells in the presence of IL-12, with activation of T-bet, STAT-1 and STAT-4 transcription factors and IFN-γ production. These cells enhance the microbicide activity of macrophages, the migration of leucocytes and the production of pro-inflammatory cytokines, promoting protection against tumor cells and intracellular microorganisms. Th2 cells, characterized by STAT-3, GATA-3 and IL-4, promote an immune response against extracellular parasites. Th17 cells are involved in eliminating extracellular bacterial and fungal pathogens and are classified by RORc and IL-17. T regulatory (Treg) cells control the immune response against self and non-self-antigens, inflammation, autoimmune diseases, allergy, asthma and pathogen-induced immunopathology, and feto-maternal tolerance. The main markers of Treg cells are CD25, CTLA-4, GITR, LAG-3, CD127, FoxP3, TGF-β and IL-10 [14-16].

The modulation of the immune response has been an approach for treating several diseases, and natural products have been investigated for their immunomodulatory action [17-19]. Our research group has been studying propolis for almost 30 years [1, 2, 6, 20].

Propolis effects on APCs and other cells involved in the immune response have been documented [11, 21-23]. Here, we sought to advance in the knowledge about propolis immunomodulatory effects on DCs and T cells, assuming that it may modulate antigen presentation and T lymphocytes activation. Melanoma-associated antigen 1 (MAGE-1) present in melanoma and other tumors [24] was used as an antigen, leading to Th1 cells activation. Retinoic acid (RA), a vitamin A metabolite, promotes expansion of human Tregs *in vitro* and prevents them from converting to Th1 or Th17 cells, sustaining Foxp3 and other Treg-related markers and their suppressive action [25]. Lymphocyte proliferation, transcription factors activation (T-bet and FoxP3) and cytokine production by DCs (TNF-α, IL-10, IL-6 and IL-1β) and T lymphocytes (IFN-γ and TGF-β) were analyzed, in order to investigate whether propolis could drive preferentially to a differential activation profile such as Th1 or Treg.

## Methods

### Propolis, MAGE-1, retinoic acid, and combinations

Green propolis was produced by Africanized honeybees (*Apis mellifera* L.) in the Beekeeping Section (UNESP, Campus Botucatu, Brazil) and kept at -20°C. The same sample has been used in all assays performed by our group, preparing fresh extracts. Its composition was analyzed by gas chromatography-mass spectrometry (GC-MS) [26]; in addition, a new chromatographic analysis of the same frozen sample was performed years later, demonstrating no effect of time and freezing on its chemical composition [27].

Propolis was ground and 30% ethanolic extracts were prepared using 70% ethanol [28]. Its dry weight was calculated (110 mg/mL). Propolis was diluted in RPMI 1640 (Cultilab, Brazil) supplemented with 10% fetal bovine serum (FBS) to obtain 5 μg/mL. 

Human MAGE-1 (Enzo Life Science, USA) was diluted in RPMI 1640 to obtain 10 μg/mL. RA (Cayman Chemical, USA) was diluted in dimethyl sulfoxide (DMSO) and then in RPMI to obtain 10^-7^ M.

The combinations of propolis with MAGE-1 and RA were prepared according to previous standardization in our laboratory.

### Healthy blood donors and monocyte isolation

Venous blood was obtained from five healthy volunteers’ donors (aged between 20 and 40 years, both genders, non-smokers, not sick or using any type of medication) and centrifuged using Ficoll-Paque (GE Healthcare Bio-Sciences, Sweden) to obtain the peripheral blood mononuclear cells (PBMC). All subjects signed an informed consent for the study, which was approved by the Ethics Committee of Botucatu Medical School, UNESP (CAAE: 42600915.0.0000.5411). 

Monocytes and lymphocytes were isolated by the negative magnetic selection technique “MACS: *magnetic-activated cell sorting*” (Miltenyi Biotec Inc., USA). Monocytes were used immediately for DCs differentiation and lymphocytes were cryopreserved in RPMI containing 10% FBS + 10% DMSO and stored in liquid nitrogen.

### CD14^+^ and CD4^+^ T cells phenotyping

CD14^+^ and CD4^+^ cells were transferred to cytometry tubes (BD Becton Dickinson and Company, USA) and centrifuged at 650 *g* for 10 min. After discarding the supernatant, cells were incubated with monoclonal antibodies (mAbs - Biolegend, USA) anti-CD14 conjugated with PerCP-CY5.5 and anti-CD4 conjugated with PerCP-CY5.5 (0.3 μL) for 30 min. A control tube (autofluorescence) with no labeled cells and an isotypic control tube were included in each test. Cells were analyzed in a flow cytometer model FACS CaliburTM (BD Becton Dickinson and Company, USA), acquiring 50.000 events.

### DC generation and phenotyping

DCs were generated from monocytes isolated from PBMC. Purified monocytes (1 × 10^6^ cells/mL) were resuspended in RPMI 1640 containing human recombinant IL-4 (80 ng/mL) and GM-CSF (80 ng/mL) (R&D Systems, USA) for 7 days at 37°C and 5% CO_2_ [29, 30]. Then, cells were incubated with mAbs (Biolegend, USA) anti-CD14-PerCP-Cy 5.5 (0.3 μL), anti-CD1a-FITC (1 μL), anti-CD83-PE (1 μL) and anti-CD11c-APC (1 μL) for 30 min. A Fluorescence Minus One (FMO) control was included. 

This phenotyping protocol was performed to assure the cell differentiation and analyzed in a flow cytometer model FACS CaliburTM (BD Becton Dickinson and Company, USA). A total of 50.000 events were acquired and the expression of following cell surface markers was analyzed: CD14^low^/CD1a^high^/CD11c^high^/CD83^low^ [31]. 

DCs were incubated with propolis alone or in combination with MAGE-1 and RA for 48 h. 

### Cell viability

Cell viability was performed using the 3-(4,5-dimethyl-thiazol-2-yl)-2,5diphenyltetrazolium bromide (MTT - Sigma-Aldrich, USA) colorimetric assay. 

DCs were incubated with the stimuli in a final volume of 100 μL. Supernatants were removed and 100 μL of MTT (1 mg/mL) were added to the culture cells. After 3 h, MTT was removed and 100 μL of DMSO (Sigma-Aldrich, USA) was added to dissolve the formazan salt. The absorbance was recorded at 540 nm and the percentage of cell viability was calculated using the formula: [(OD test/OD control) x 100].

### Cytokine production by DCs

In an attempt to investigate propolis modulatory effects, the production of pro- and anti-inflammatory cytokines was analyzed after DCs incubation with the stimuli. The supernatants were harvested from the cell cultures for TNF-α, IL-6, IL-1β and IL-10 quantitation by enzyme-linked immunosorbent assay (ELISA) according to the manufacturer’s instruction (R&D Systems, USA). Lipopolysaccharide 1 µg/mL (isolated from *Escherichia coli* O26:B6 - Sigma-Aldrich, USA) was used as a positive control. The absorbance was determined at 450 nm using a microplate reader (ELx800, BioTek, Germany).

### T CD4^+^ cell proliferation

Isolated CD4^+^ cells were labeled with carboxy-fluorescein succinimidyl ester (CFSE) (Cell-Trace CFSE Proliferation Kit, Molecular Probes, Invitrogen, USA) to monitor lymphoproliferation. For the co-culture assays, DCs incubated with MAGE-1 or RA simultaneously or not with propolis for 48 h were incubated with CFSE-labeled autologous CD4^+^ T lymphocytes (ratio DCs/lymphocytes = 1/10) for 120 h. Phytohemagglutinin (PHA - 2.5 μg/mL) was used as a positive control for cell proliferation and cells without any marking (autofluorescence) were used as a negative control, in addition to FMO control under the same conditions. After incubation, the lymphocyte proliferation was evaluated in a flow cytometer model FACS CaliburTM (BD Becton Dickinson and Company, USA), and a total of 50.000 events were acquired.

### Transcription factor gene expression

T-bet and FoxP3 expression by T CD4+ cells was evaluated by real-time quantitative reverse transcription polymerase chain reaction (RT-qPCR), using the 7300 Real Time PCR System (Applied Biosystems, USA). 

After treating DCs with propolis alone or in combination with the stimuli by 48 h, cells were incubated with lymphocytes by 120 h. The total RNA was extracted from lymphocytes using the RNeasy Mini Kit (Qiagen, The Netherlands) and treated with RQ1 RNase-Free DNase (Promega, USA). cDNA synthesis was performed using the ProtoScript II Reverse Transcriptase kit (BioLabs, USA). The GoTaq-qPCR Master Mix (Promega, USA) was used and Table 1 presents the primers sequence. Each reaction was performed in triplicate and the conditions were: 50°C/2 min, 95°C/10 min for initial denaturation, 40 cycles at 95°C/15s and 60°C/60s followed by the melting curve. 

The expression values ​​of the transcripts were normalized using the glyceraldehyde-3-phosphate dehydrogenase (GAPDH). The differential expression of the selected genes was performed using a standard-curve [32]. All samples were standardized in relation to an RNA sample using a relative value of 100. 

**Table 1.** Sequence of primers for the transcription factors and GAPDH.

Genes	Sequence (5’-3’)	GeneBank
*T-bet*	Forward primer: (906) GGATGCGCCAGGAAGTTTCA (925) Reverse primer: (993) TGGAGCACAATCATCTGGGT (974)	NM_013351
*FoxP3*	Forward primer: (614) AGGAAGGACAGCACCCTTT (633) Reverse primer: (726) GGAAGTCCTCTGGCTCTTCG (707)	NM_014009
*GAPDH*	Forward primer: (684) CGTGGAAGGACTCATGACCA (703) Reverse primer: (801) GGCAGGGATGATGTTCTGGA (782)	NM_002046.4

### Intracytoplasmic cytokine analysis

Six hours before ending the incubation of the co-cultures, cells were treated with brefeldin A (Biolegend, USA) in order to prevent the release of cytokines from the cell cytoplasm.

Cells were labeled with anti-CD4 conjugated to PerCP/Cy5.5 (OKT4 clone - Biolegend, USA) which allowed the selection of the gate of only the CD4+ lymphocytes and, for Treg cells, with anti-CD25 conjugated with APC (clone M-A251 - Biolegend). Cells were incubated for 30 min in the dark at 4°C and then centrifuged for 10 min at 650 *g*. After, the supernatant was discarded and cells were incubated for 15 min with 100 μL of the solution A of Fix & Perm Cell Fixation and Permeabilization kit (Nordic MUbio, The Netherlands). After washing with ISOTON, cells were centrifuged at 605 *g* for 10 min, the supernatant was discarded and 100 μL of solution B of the *kit Fix & Perm* containing anti-IFN-γ conjugated with PE (clone B27 - Biolegend) and anti-TGF-β1 conjugated with PE (clone TW4-2F8 - Biolegend). After incubation, the cells were analyzed by flow cytometry and, for each test, an isotypic control with the respective test fluorochromes, an autofluorescent control and FMO controls were included. The analyses were performed using the flow cytometer model FACS CaliburTM (BD Becton Dickinson and Company, USA) and the FlowJo software vX.0.7. 50.000 acquisition events were standardized per sample and the population of interest was optimized by establishing a gate based on size (FSC) and granularity (SSC) parameters. The results were expressed as the percentage of CD4 positive cells expressing IFN-γ or TGF-β1.

### Statistical analysis

Data were analyzed using the Graph Pad statistical software (Graph Pad Prisma, USA). Analysis of variance (ANOVA) and Dunnett’s test were employed (*p* < 0.05). Data were expressed as the mean ± standard deviation of 5 individuals. A *p* value of less than 0.05 was considered significant.

## Results

### DC phenotyping and viability

**Figure 1.**
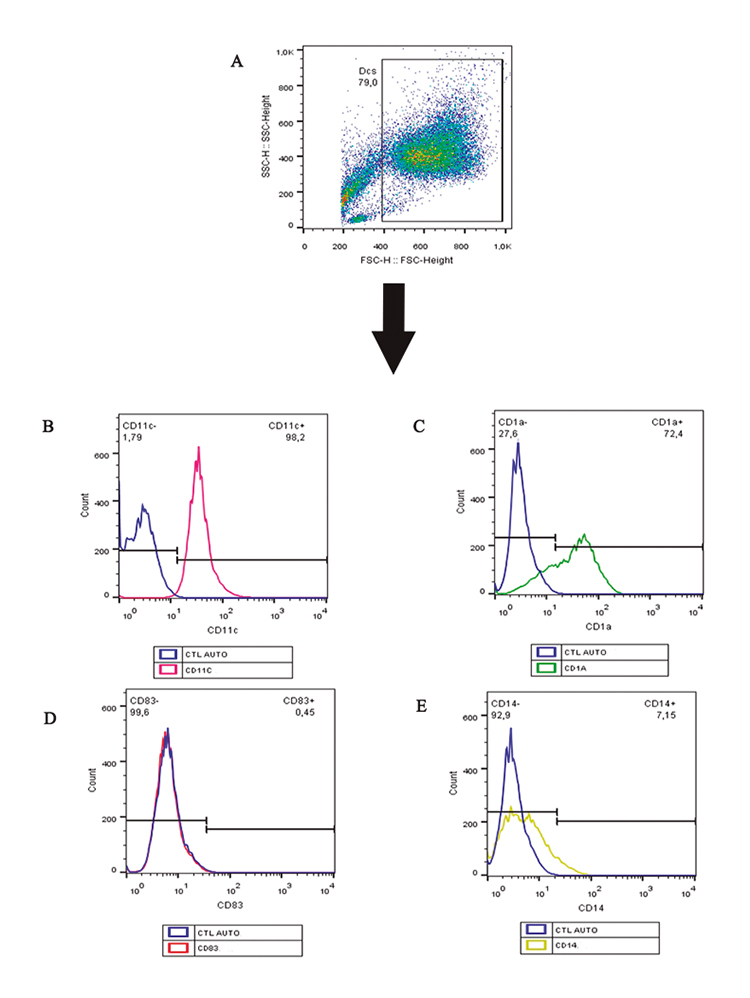
Dendritic cell phenotype after monocyte incubation with IL-4 and GM-CSF. **(A)** Dot plot related to size (FSC-H) x granularity (SSC-H). Histograms represent cell surface markers: **(B)** CD11c^high^, **(C)** CD1a^high^, **(D)** CD83^low^ and **(E)** CD14^low^.

DCs were properly generated from monocytes, presenting the typical cell markers CD11c^high^, CD1a^high^, CD83^low^ and CD14^low^ (Figure 1).

**Figure 2.**
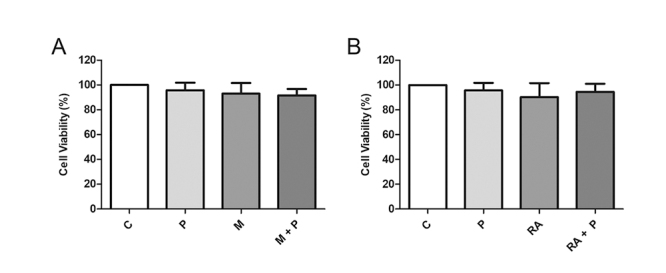
Viability (%) of dendritic cells (1 × 10^6^cells/mL) after 48 h incubation with RPMI 1640 (control - C), propolis (P - 5 μg/mL), **(A)** MAGE-1 (M - 10 μg/mL), **(B)** retinoic acid (RA - 10^-7^M) and their combination. Data represent mean and standard deviation of five subjects (*p* > 0.05).

To verify a possible cytotoxic effect, DCs were incubated with propolis and the stimuli (MAGE-1 and RA) simultaneously or not and cell viability was assessed (Figures 2A and 2B). Neither the treatments nor the solvents (propolis: 70% ethanol - 0.013%; RA - DMSO 0.0002%) affected cell viability (data not shown).

### Cytokine production by DCs

**Figure 3.**
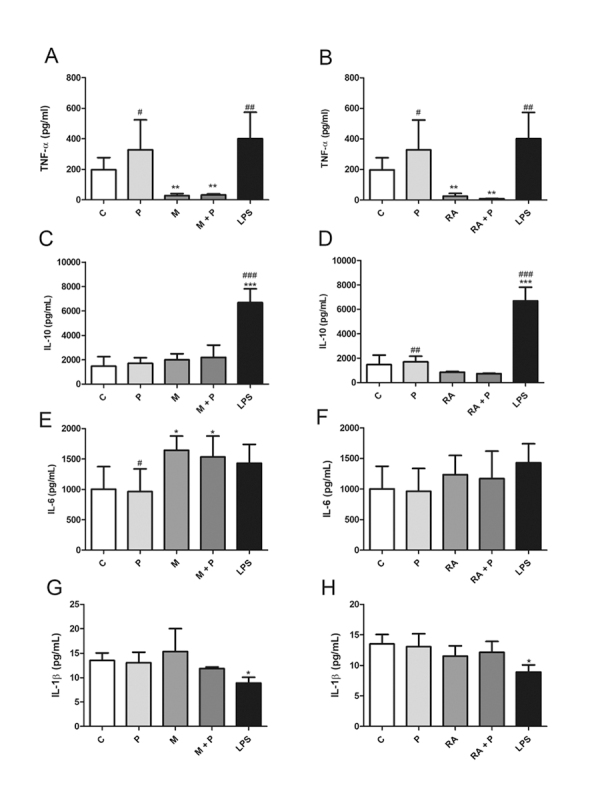
Cytokine production (pg/mL) by dendritic cells (1 × 10^6^cells/mL) after 48 h incubation with RPMI 1640 (control - C), propolis (P - 5 μg/mL), **(A, C, E, G)** MAGE-1 (M - 10 μg/mL), **(B, D, F, H)** retinoic acid (RA - 10^-7^M), their combination, and LPS (1 µg/mL). Data represent mean and standard deviation of five subjects. Significantly different from control: *(*p* < 0.05); **(*p* < 0.01); ***(*p* < 0.001). Significantly different from the respective combination: ^#^(*p* < 0.05); ^##^(*p* < 0.01); ^###^(*p* < 0.001).

MAGE-1 and RA alone or in combination with propolis inhibited TNF-α production by DCs compared to control (Figures 3A and 3B, respectively). 

MAGE-1 + propolis seemed to induce slightly IL-10 production, although not significantly (Figure 3C). RA alone or in combination with propolis exerted no effect on IL-10 (Figure 3D).

MAGE-1 alone or in combination with propolis induced IL-6 production, while RA did not affect it (Figures 3E and 3F). 

No differences were seen in IL-1β production; however, MAGE-1 showed a tendency to increase it, whereas the combination with propolis maintained IL-1β levels similar to control (Figures 3G and 3H).

### T lymphocyte proliferation

**Figure 4.**
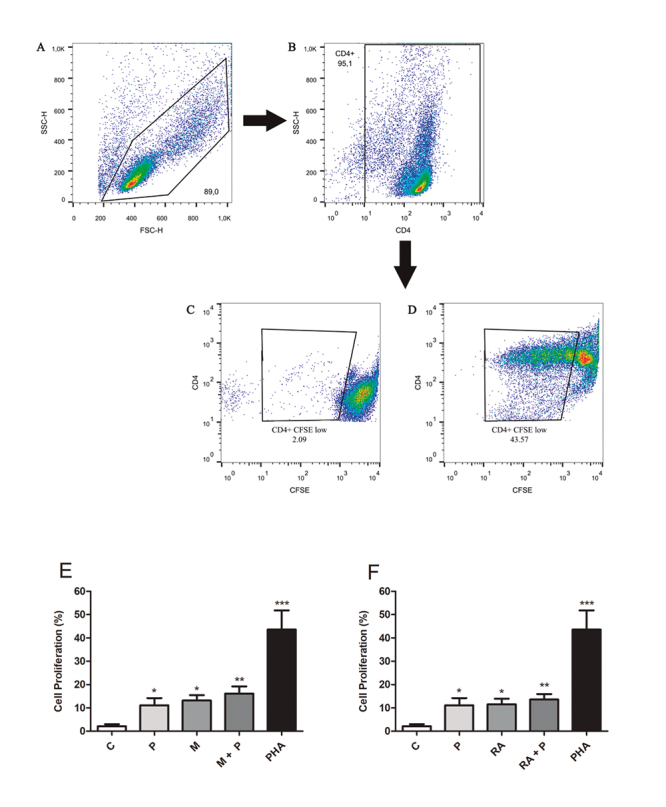
Representative dot plots of lymphocyte proliferation after 120 h of co-culture with autologous dendritic cells. **(A)** Gate of lymphocytes by size (FSC-H) x granularity (SSC-H). **(B)** Gate of CD4^+^ cells. **(C)** Proliferation of control lymphocytes (cells incubated with RPMI). **(D)** Proliferation of lymphocytes incubated with the positive control (PHA - 2.5 μg/mL). Percentage (%) of lymphocytes (1 × 10^6^cells/mL) proliferation after 120 h of co-culture with autologous dendritic cells treated only with RPMI 1640 (control - C), PHA (2.5 μg/mL), propolis (P - 5 μg/mL), **(E)** MAGE-1 (M - 10 μg/mL), **(F)** retinoic acid (RA - 10^-7^M) and their combination for 48 h. Data represent mean and standard-deviation (n = 5). Significantly different from control: *(*p* < 0.05); **(*p* < 0.01); ***(*p* < 0.001).

A possible influence of propolis and stimuli on lymphoproliferation was assessed. Representative Dot Plots of lymphocyte proliferation are shown in Figure 4 (panels A, B, C and D). A higher percentage of proliferation was seen after co-culture of lymphocytes with DCs treated with propolis, MAGE-1 and RA, alone or in combination compared to untreated DCs (Figures 4E and 4F).

### Transcription factor expression and cytokine production

**Figure 5.**
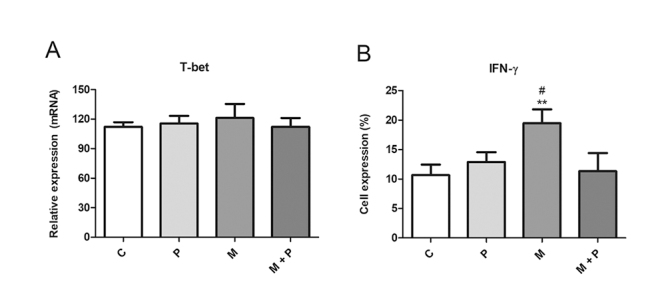
(A) T-bet relative expression and (B) percentage (%) of lymphocytes expressing IFN-γ after 120 h of co-culture with autologous dendritic cells treated with RPMI 1640 (control - C), MAGE-1 (M - 10 μg/mL), propolis (P - 5 μg/mL) or their combination. Data represent mean and standard deviation (n = 5). Significantly different from control: **(*p* < 0.01). Significantly different from M + P: ^#^(*p* < 0.05).

Since transcription factors and cytokines are signatures of T cell subsets, we analyzed T-bet mRNA levels and the percentage of lymphocytes expressing IFN-γ, to observe the effects of propolis and stimuli on the differentiation of Th1 cells. Propolis and MAGE-1 did not affect T-bet expression (*p* > 0.05) (Figure 5A). MAGE-1 induced IFN-γ production by T CD4+ cells (*p* < 0.01), while propolis led it to basal levels (Figure 5B).

**Figure 6.**
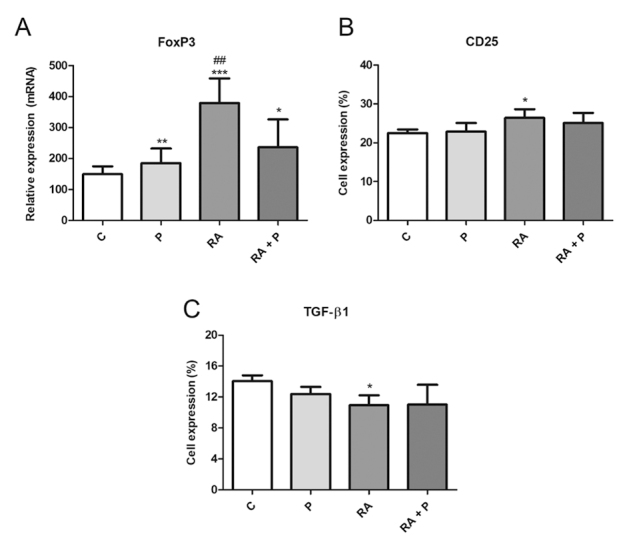
(A) FoxP3 relative expression and percentage (%) of lymphocytes expressing (B) CD25 and (C) TGF-β1 after 120 h of co-culture with autologous dendritic cells treated with RPMI 1640 (control - C), retinoic acid (A - 10^-7^ M), propolis (P - 5 μg/mL) or their combination. Data represent mean and standard deviation (n = 5). Significantly different from control: *(*p* < 0.05), **(*p* < 0.01), ***(*p* < 0.001). Significantly different from RA + P: ^##^(*p* < 0.01).

To evaluate the activation status of Treg cells, CD25 and FoxP3 were examined. TGF-β1 was analyzed to verify the functionality of the cell population in our culture. RA induced FoxP3 and CD25 expression (Figures 6A and 6B, respectively) and inhibited TGF-β1 production (Figure 6C). Propolis alone or in combination with RA stimulated FoxP3 expression (Figure 6A).

## Discussion

DCs are professional APCs, linking innate and adaptive immunity with the additional activation of naïve T lymphocytes and determining the balance between Th1, Th2, Th17 and Treg cells [33]. Monocyte-derived DCs are an interesting model to investigate the function of DCs *in vitro* [34]. GM-CSF and IL-4 lead to the differentiation of monocytes in DCs with immature phenotype expressing high levels of CD11c and CD1a, and declining the levels of adhesion molecules as LFA-1, ICAM-1 and LFA-3, class II major histocompatibility complex (HLA-DR), co-stimulatory molecules (CD40, B7-1/CD80, B7-2/CD86) and CD14 [35].

Monocytes were properly differentiated into immature DCs and treated with propolis, MAGE-1 and RA, which did not affect cell viability. Likewise, other studies have shown that propolis or its compounds exert no cytotoxic action on DCs [23, 36, 37].

Regarding the innate immunity and the production of pro-inflammatory cytokines by DCs, MAGE-1 and RA alone or in combination with propolis inhibited its production. MAGE-1 showed a tendency to increase IL-1β production, while the combination with propolis maintained basal levels. Propolis and the stimuli did not affect IL-6 production. The combination propolis + MAGE-1 showed a tendency to increase the production of the anti-inflammatory cytokine IL-10. In agreement with these findings, propolis exerted an anti-inflammatory action in the production of cytokines by human monocytes, decreasing TNF-α and IL-6 levels in combination with MAGE-1 and RA, and increasing IL-10 production in combination with MAGE-1, what indicated that propolis potentially affected innate immunity by downmodulating the pro-inflammatory activity of monocytes [38].

Besides the specific nature of each antigen, the role of DCs driving the immune response is essential to define the signals that will be communicated to naïve T lymphocytes, inducing apoptosis, anergy, tolerance or activation of Th1, Th2, Th17 or a Treg profile [39]. Regarding adaptive immunity, our findings are in agreement with these authors, who evaluated MAGE-3 and the activation of Th1, Th2 or Th17 profiles. After DCs incubation with this antigen, a strong polarization was seen towards the Th1 profile.

There was a higher proliferation of lymphocytes after coculture with DCs treated with propolis, MAGE-1 and RA than control. In contrast, 9-cis RA (a RA derivative) inhibited the lymphoproliferation induced by DCs, associated to reduced IFN-y levels [40].

MAGE-1 and MAGE-3 are clinically relevant antigens expressed in human melanomas and other tumors, but not in normal tissues except testis [24]. Here, MAGE-1 increased IFN-y production but this effect was prevented by propolis, suggesting that it may inhibit the Th1 profile. In fact, Okamoto et al. [41] demonstrated that murine spleen cells treated with Brazilian propolis inhibited the generation of Th1 cells, reducing T-bet expression and IFN-y production. Additionally, BALB/c mice fed with propolis after induction of colitis had a lower Th1 cell-mediated inflammatory response and low IFN-y levels. 

Inhibition of Th1 profile may be associated with an impaired antitumoral immune response. Nonetheless, other cells may attack tumor cells, such as natural killer cells, macrophages and T CD8+ lymphocytes. Mice with metastasis treated with propolis exhibited activation of T CD8+ cells, suggesting its effect on the antitumoral immune response. The antitumor activity of propolis *in vivo* may be associated to its immunomodulatory effect and the activation of macrophage and T CD8+ cells [42, 43].

On the other hand, Th1 cells may exert an inflammatory response causing a pathologic condition as observed in autoimmune diseases. Our findings and those of the literature highlight the potential of propolis in controlling inflammatory processes.

Treg cells exert a critical role in inducing and maintaining the peripheral tolerance and antigen-induced inflammation. These cells are typically immunosuppressive due to the production of TGF-β and IL-10, blocking T cell activation and function. TGF-β suppresses target cells while IL-10 inhibits the activation of APCs and the effects of IFN-y, controlling inflammatory responses [15, 44, 45]. RA is a vitamin A metabolite that impairs the conversion of Treg cells into a Th1 or Th17 profile, maintaining FoxP3 expression. Propolis induced Foxp3 expression without affecting CD25 expression and TGF-β production. Propolis + RA induced the expression of Foxp3 and slightly that of CD25 nonsignificantly. RA inhibited TGF-β production, which was counteracted by propolis. This indicates that propolis leads to the activation of a regulatory profile, which has been observed both *in vitro* and *in vivo* [7, 8, 41]. 

Treg cells play an important role in infectious diseases, tumors and periodontitis. In HIV-infected patients, disease progression is directly related to immune hyperactivation, and in these cases there is a reduction in the number and function of Treg cells. On the other hand, studies with Treg cells in malignant neoplasms suggested that the increased activity of these cells is associated with an impaired antitumor immune response. Thus, inhibition of Treg cell function could have positive results as a therapeutic strategy for the treatment of cancer [46]. Regarding periodontitis, Cafferata et al. [47] reported that an approach for treating periodontitis would be an increase in the number of Treg cells or in the levels of anti-inflammatory cytokines such as IL-10 and TGF-β1 produced in part by these cells.

The interest in the therapeutic applications of propolis is expressive [2, 4, 48, 49] and research has advanced considerably to discover its main mechanisms of action. Although it is still difficult to obtain a universal standardization, the analysis of its chemical composition has revealed interesting molecules with immunomodulatory action [1]. Propolis samples produced in the south of Brazil under organic conditions were grouped in seven types according to chromatographic methods, which seemed to be a source of bioactive compounds with antioxidant, antibacterial and anti-inflammatory action [50]. Here, a properly characterized propolis sample was used and its main compounds were flavonoids (kaempferid, 5,6,7-trihydroxy-3,4’-dimethoxyflavone, aromadendrine-4’-methyl ether); essential oils (spathulenol, (2Z,6E)-farnesol, benzyl benzoate and prenylated acetophenones); aromatic acids (dihydrocinnamic acid, *p*-coumaric acid, ferulic acid, caffeic acid, 3,5-diprenyl-*p*-coumaric acid, 2,2-dimethyl-6-carboxy-ethenyl-8-prenyl-2H-1-benzo-pyran); a prenylated *p*-coumaric acid and two benzopyranes: *E* and *Z* 2,2-dimethyl-6-carboxyethenyl-8-prenyl-2H-benzopyranes); di- and triterpenes, among others. Furthermore, investigating the same propolis sample in our research allows us to propose mechanisms of action displayed by this sample.

Previous findings of our group revealed that propolis induced TLR-4 expression, NF-kB pathway, TNF-α, IL-6 and IL-10 production, increasing DCs bactericidal activity [23]. Here, propolis-treated DCs stimulated lymphocyte proliferation and led to Th1 and Treg profiles. Although it is difficult to precisely indicate which constituents of propolis may be involved in our findings, it is likely that phenolic acids (caffeic, dihydrocinnamic and *p*-coumaric acids) stimulated DCs, as they participated in the stimulating action of propolis in monocytes [51]. In addition, previous findings of our group revealed that propolis constituents act by binding to TLR-2 and TLR-4, since some biological activities displayed by monocytes were affected by blocking such receptors [52].

Evidence points to the potential of propolis and its constituents for the development of new anti-inflammatory drugs, inhibiting cytokines, intracellular signaling pathways, cell adhesion and migration [12]. Constituents from Brazilian green propolis such as baccharin exerted an anti-inflammatory action by inhibiting the production of cytokines and eicosanoids in mice, while *p*-coumaric acid also stimulated IL-10 production [53]. In a clinical trial, propolis increased Foxp3 expression by lymphocytes in HIV-infected people exhibiting a previous inflammatory status [4]. Our findings have practical applications and indicate that propolis should be further investigated *in vivo* to control inflammatory and autoimmune diseases, and pathogen-induced immunopathology. Propolis isolated compounds should be evaluated in clinical trials as well.

## Conclusions

Together, our data revealed that propolis modulates DC and T cell functions, indicating that the *in vitro* model using MAGE-1 and RA-treated DCs seemed to be feasible to affect Th1 and Treg cells subsets. These findings are unprecedented and relevant, revealing propolis potential to treat inflammatory conditions such as autoimmune diseases and pathogen-induced immunopathology.
